# Optimizing skill acquisition: the role of self-assessment during a continuous training program for laparoscopic suturing

**DOI:** 10.1007/s00464-025-11682-9

**Published:** 2025-03-24

**Authors:** Vera Hillemans, Daan J. Verhoeven, Xander van de Mortel, Guus M. J. Bökkerink, Sanne M. B. I. Botden, Maja Joosten, Ivo de Blaauw

**Affiliations:** 1 Department of Surgery, Radboud University Medical Center (Radboudumc), Geert Grooteplein Zuid 10, Route 618, 6500HB Nijmegen, The Netherlands; 2 Radboudumc – Amalia Children’s Hospital, Nijmegen, The Netherlands; 3 Department of Pediatric Surgery, Radboudumc – Amalia Children’s Hospital, Nijmegen, The Netherlands; 4 Princess Máxima Center for Pediatric Oncology, Utrecht, The Netherlands

**Keywords:** Laparoscopy, Surgical training, Self-assessment, Laparoscopic suturing, Performance, Medical education

## Abstract

**Background:**

Mastering laparoscopic suturing, a critical aspect of minimally invasive surgery, remains a challenge. Self-assessment and ‘reflection before practice’ may enhance the learning curve of laparoscopic suturing. This study investigates the optimal frequency of self-assessment and reflection before practice in laparoscopic suturing training.

**Methods:**

Participants (medical students, surgical residents, and medical PhD students) underwent laparoscopic suturing training at home using a laparoscopic simulator (LaparoscopyBoxx). Three groups were formed: a ‘control group’ without self-assessment, a ‘periodic self-assessment group’, and a ‘continuous self-assessment group’. The validated Laparoscopic Suturing Competency Assessment Tool (LS-CAT) served as self-assessment form. Participant’s performance was quantified by objective parameters (time, distance, handedness, off-screen time, speed, acceleration, smoothness and distance between the instruments) measured by SurgTrac software.

**Results:**

No significant differences were observed between groups for primary outcome parameters time and distance across tests. However, significant differences emerged in secondary outcome parameters off-screen (baseline-test, *p* = 0.018), acceleration (baseline-test, *p* = 0.007), smoothness (baseline-test, *p* = 0.004; after-test, *p* = 0.038) and speed (after-test, *p* = 0.033) at various tests, favoring the self-assessment groups.

**Conclusion:**

Self-assessment and reflection before practice may lead to more efficient instrument utilization and increased safety. A lower frequency of self-assessment and reflection before practice offered comparable benefits, which optimizes training efficiency, and is therefore recommended.

## Introduction

Laparoscopic surgery has progressed significantly over the past decades and is now considered a standard approach for both elective and acute procedures [1, 2]. The demand for surgeons able to perform increasingly complex minimally invasive procedures is growing, so there is a need for surgeons to refine these complex technical skills. The numerous benefits for patients of minimally invasive techniques include reduced postoperative pain, shorter hospitalization, and faster recovery [3, 4]. Laparoscopic suturing constitutes a crucial component of laparoscopic surgery, while mastering these specific skills proves to be challenging [5–8]. The success of laparoscopic procedures depends on the surgeon's skill, particularly in the realm of suturing techniques [9, 10]. The complexity of suturing, limits further expansion of laparoscopic surgery [7].

The ability to perform accurate and effective sutures during laparoscopic procedures requires a combination of technical skills, spatial awareness, and hand–eye coordination [11, 12]. For the surgeon’s ongoing development, self-evaluation is crucial. It enables skills enhancement through critical reflection and continuously addresses areas for improvement [13, 14]. Despite the complexity of laparoscopic suturing, research has demonstrated that practice can significantly improve suturing skills, even conducted in a home-based setting [15]. Moreover, prior research underscores the added value of self-assessment in mastering minimally invasive suturing [14, 16]. The use of self-assessment can lead to a higher skills level at the start of the learning curve [17]. This study aims to determine the optimal frequency of self-assessment in laparoscopic suturing, addressing a current gap in literature and enhancing effectiveness of surgical training.

## Methods

### Participants

Medical students, junior doctors working as physicians in a surgical department and medical PhD students conducting research within the field of surgery were included, in the period from May 2020 until August 2021. Only medical students who started their internships and junior doctors who were not in training to become a surgeon yet, were included. All participants were recruited at the Radboud University Medical Center, Nijmegen, and at University Medical Center Utrecht, the Netherlands.

All participants were categorized into three groups: ‘control group’ without self-assessment, ‘periodic self-assessment (SA) group’ and ‘continuous self-assessment (SA) group’. All groups followed the identical training schedule, as outlined in the chapter ‘Protocol’. The control group did not use self-assessment and reflection before practice. In contrast, participants in the periodic SA group and continuous SA group, studied the self-assessment form beforehand as a ‘reflection before practice’ and completed the form afterwards. The periodic SA group exclusively applied reflection before practice and self-assessment during the three test-moments, while the continuous SA group performed this during every training session and all test moments.

### Equipment

**Fig. 1:**
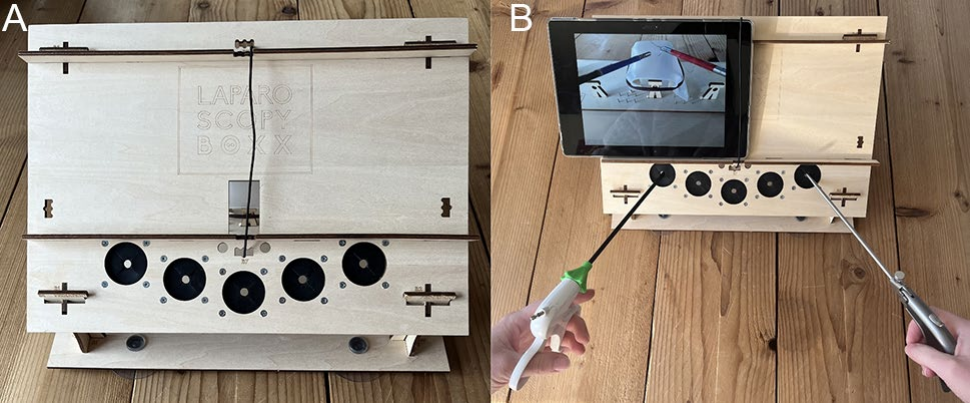
LaparoscopyBoxx

For this study the low budget wooden box trainer LaparoscopyBoxx by Outside the Boxx [18, 19] was used, as shown in Fig. 1. This simulation trainer for minimal invasive surgery consisted of a wooden box with three to five instrument ports. A tablet camera (or smartphone) can be positioned through an opening on the top of the wooden box. This allows the tablet to function as laparoscopic simulation screen. In this study a Lenovo P10 tablet (Digital River Ireland Ltd) was used [20].

**Fig. 2:**
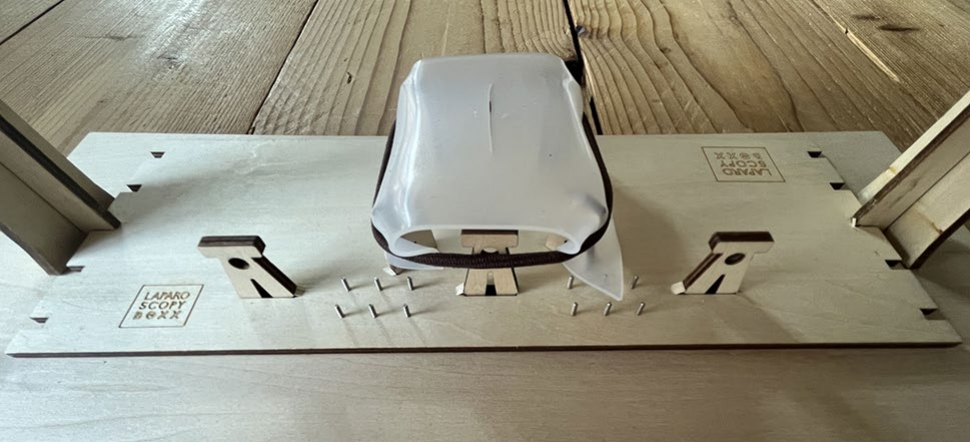
Exercise board inside the LaparoscopyBoxx

The utilized instruments included a 5 mm laparoscopic needle holder, a grasper (curved (Maryland dissector) or straight) and scissors. The inside of the wooden box contained an exercise board with a wooden framework, on which a suturing pad was fixated with an incision in the center (Fig. 2).

On the tablet, the tracking software SurgTrac (eoSurgical Ltd., Edinburgh, United Kingdom) was installed [21, 22]. SurgTrac measured various parameters by tracking a red (right) and blue (left) marker, placed at the end of the laparoscopic instruments. The software measured the following parameters: time of executing a task (in seconds), distance traveled by the left and right marking (in meters), distance between the left and right marking (average distance between the red and blue marking in centimeters), instruments off-screen (in percentage of time), speed (mean, in millimeters/second), acceleration (mean, in millimeters /second^2^), smoothness (mean, in millimeters /second^3^) and handedness (percentage left or right instrument usage).

### Task

**Fig. 3:**
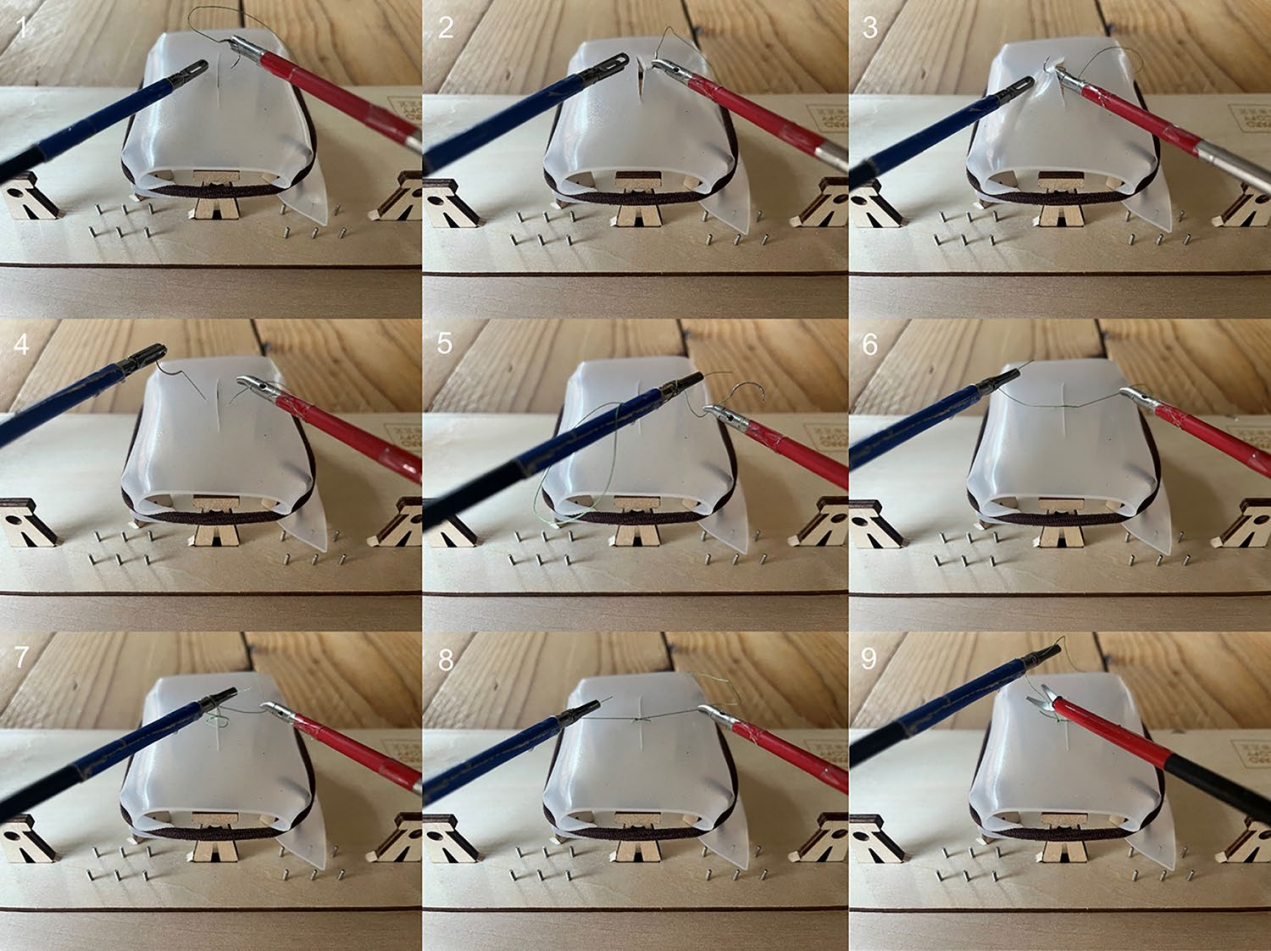
Laparoscopic suturing task, executed on a simulator

All participants performed a suturing task (Fig. 3), which involved of placing a suture on the suturing pad using an intracorporeal knot. The suturing thread with needle was positioned on the suturing pad. Participants had to pick up the needle with a Maryland dissector and correctly position it in the needle holder. Subsequently, a suture was placed in the suturing pad in one or two tempi and an intracorporeal knot was tied.

### Self-assessment form

**Fig. 4:**
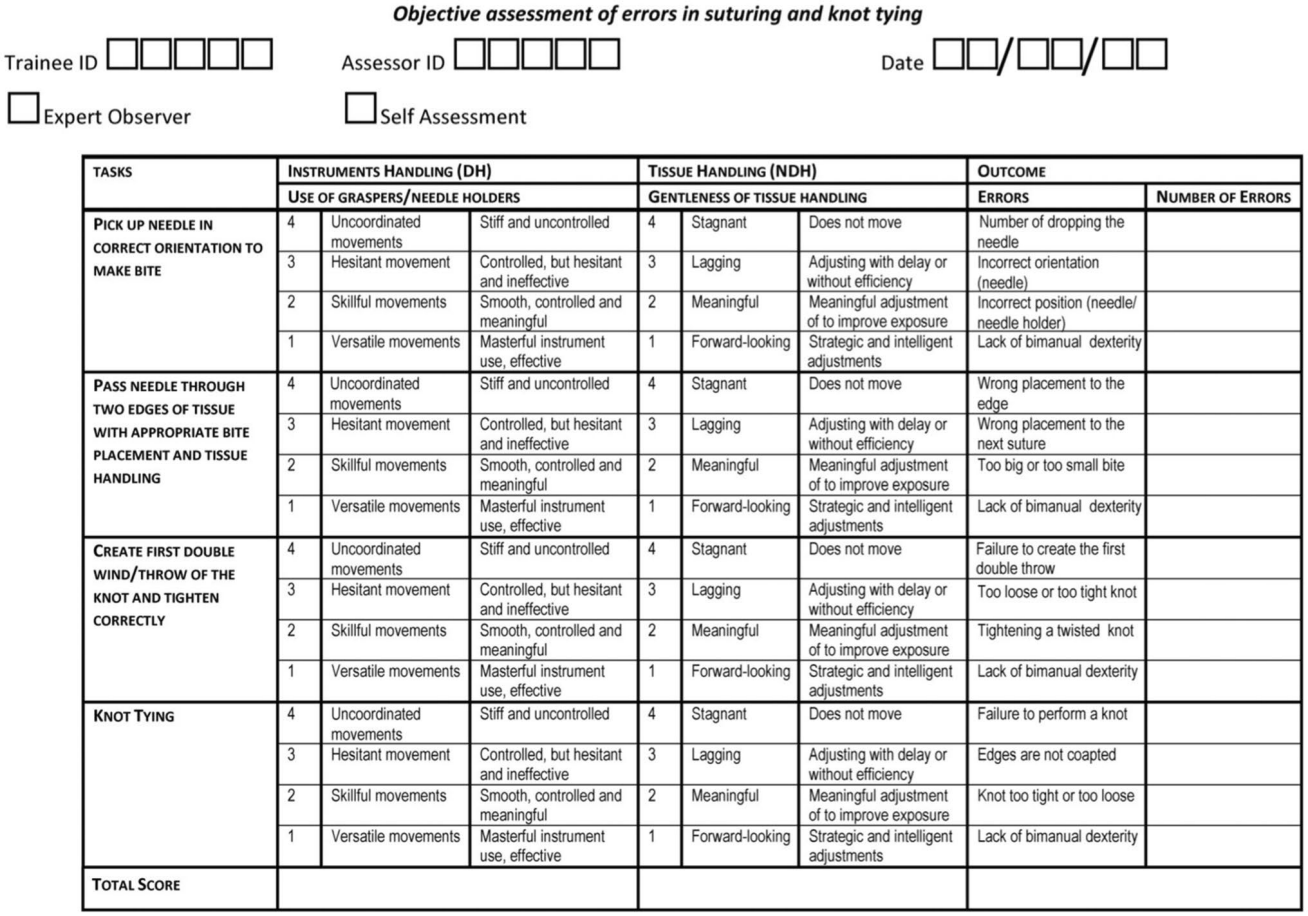
Laparoscopic suturing competency assessment tool (LS-CAT)

A Laparoscopic Suturing Competency Assessment Tool (LS-CAT, Fig. 4) functioned as the self-assessment form. This assessment tool was developed and previously validated for the objective assessment of laparoscopic suturing skills [23]. The LS-CAT consists of four sub-tasks, each rated on a scale from one to four, with one indicating the highest level of proficiency. Each sub-task was evaluated for specific skills, including ‘instrument handling’ (with the dominant hand) and ‘tissue handling’ (with the non-dominant hand). Additionally, specific errors related to each sub-task were assessed and recorded. The cumulative scores for sub-tasks related to each specific skill, along with the errors, contributed to the total LS-CAT score. A low total LS-CAT score indicated better performance.

### Protocol

After inclusion, participants provided informed consent and completed a form detailling their demographics and surgical experience. Following, participants received written and video-recorded instructions (live instructions were not possible due to COVID-19). Before starting their training scheme, each participant performed a ‘pre-test’, during which the suturing task was executed and recorded once. Participants then began an intensive two-week training scheme, followed by a two-weekly training scheme.

During the initial two weeks, participants engaged in six training sessions for 45–75 min, with the suturing task performed at least once per session. After those two weeks of intensive training, participants completed the ‘baseline-test’. Subsequently, participants continued their training, practicing once every two weeks for over 30 min, over a span of three months, with the suturing task being executed in each training session. At the end of those three months, an ‘after-test’ was conducted.

During all three test-moments (pre-test, baseline-test and after-test), instruments were tracked and task execution was recorded using SurgTrac. Afterwards, participants submitted the tracking data and recorded videos via SurgTrac to the researchers.

**Fig. 5:**
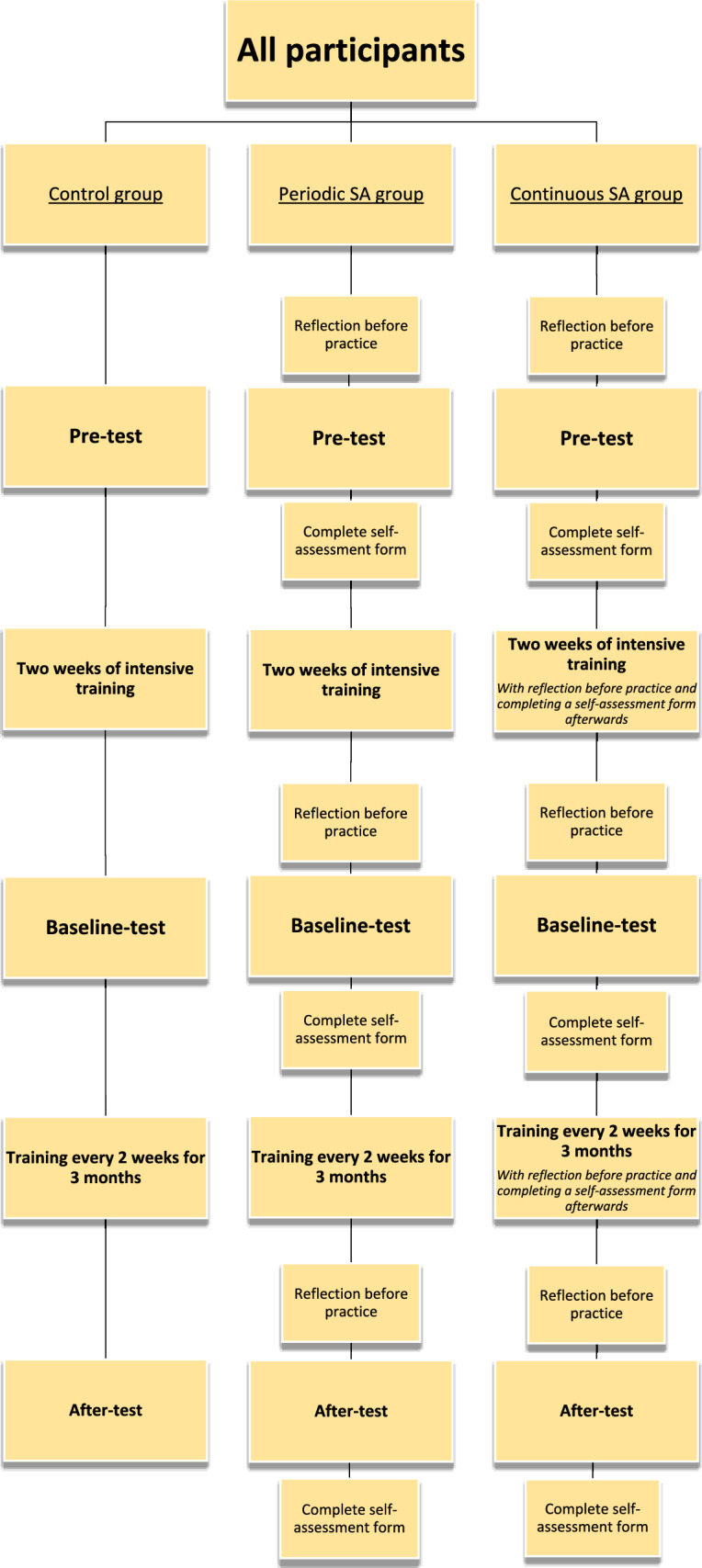
Study protocol with different training schemes

An overview of the protocol can be seen in Fig. 5.

### Outcome parameters

The primary outcome parameters were the SurgTrac parameters 'time' and 'distance', both of which have established construct validity for assessing minimally invasive surgical skills [24]. Time was defined as the total duration of task execution in seconds. Distance referred to the cumulative distance traveled by the tips of the right (red marker) and left (blue marker) instruments together, measured in meters.

The SurgTrac parameters ‘handedness’, ‘off-screen’, ‘speed’, ‘acceleration’, ‘smoothness’ and ‘distance between’ served as secondary outcome parameters. Handedness was defined as the percentage a participant differs of an equal division of right and left instrument usage. Off-screen was the average percentage of time that the instrument tips were not visible on screen. Speed was defined as the average speed of the right and left instrument in mm/s. Acceleration referred to the mean acceleration of the instruments in mm/s^2^, with lower acceleration indicating better performance. Smoothness was defined as the average smoothness of the instruments in mm/s^3^, where a lower smoothness correlated with a better performance. Distance between was the average distance between the right and left instrument tip, measured in centimeter.

### Statistical analyses

The data was analyzed using IBM Statistical Package for Social Sciences (SPSS), version 29. A *p* value < 0.05 was considered statistically significant, except for post hoc analyses using Wilcoxon signed-rank tests. In these analysis, a Bonferroni correction was applied, resulting in an adjusted significance level of *p* < 0.017. Due to the non-normal distribution of the data, Kruskall-Wallis tests were used to compare outcome parameters across different groups. Subsequent post hoc analyses were conducted with Mann–Whitney U tests. To assess differences between test-moments within groups, Friedman's analysis of variance (ANOVA) was applied. Post hoc analyses for within-group differences were performed using Wilcoxon signed-rank tests.

A sample size calculation was performed with a power of 0.80 and an alpha of 0.05. A total of 17 participants per group was needed. To account for potential drop-out, slightly more participants were included in each group.

## Results

A total of 55 participants were enrolled; nineteen in the control group, eighteen in the periodic SA group, and eighteen in the continuous SA group. The mean age of the entire cohort was 25.2 years (SD 2.7), and the majority (63.6%) was female. Among the participants were thirty-seven medical students (67.3%), twelve medical doctors (not in training, 21.8%), ten PhD candidates (18.2%), and one first-year resident (1.8%).

No significant differences were found between the groups concerning age (*p* = 0.599), gender distribution (*p* = 0.681), and profession (0.270 ≤ *p* ≤ 0.861). Additionally, reported surgical experience, encompassing observation (*p* = 0.287), assisting (*p* = 0.632) and performing (*p* = 0.557) open surgery and observation (*p* = 0.241), assisting (*p* = 0.159) and performing (*p* = 0.142) laparoscopic surgery, showed no significant differences between groups.

### Differences between groups

For the primary outcome parameter time, no significant differences were found between groups at the pre-test (control group 614.0 s, periodic SA group 692.5 s, continuous SA group 532.0 s, *p* = 0.362), baseline-test (control group 167.0 s, periodic SA group 222.0 s, continuous SA group 220.0 s, *p* = 0.222) and after-test (control group 178.0 s, periodic SA group 222.0 s, continuous SA group 221.0 s, *p* = 0.095). For the primary outcome parameter distance, no significant differences were found between groups as well at the pre-test (control group 21.1 m, periodic SA group 20.2 m, continuous SA group 23.7 m, *p* = 0.934), baseline-test (control group 7.6 m, periodic SA group 7.1 m, continuous SA group 8.1 m, *p* = 0.923) and after-test (control group 12.3 m, periodic SA group 7.6 m, continuous SA group 6.8 m, *p* = 0.468).

In addition, the pre-test revealed no significant differences between groups for all secondary outcome parameters (0.078 ≤ *p* ≤ 0.934).

At the baseline-test, a significant difference emerged for off-screen percentage between the control group and continuous SA group (control group 0.7%, continuous SA group 0.0%, *p* = 0.007). Additionally, a significant better acceleration was observed in the periodic SA group and continuous SA group compared to the control group (control group 5.6 mm/s^2^, periodic SA group 3.1 mm/s^2^, continuous SA group 3.6 mm/s^2^, 0.004 ≤ *p* ≤ 0.013). The periodic SA group and continuous SA group also scored a better smoothness, compared to the control group (control group 0.3 mm/s^3^, periodic SA group 0.1 mm/s^3^, continuous SA group 0.1 mm/s^3^, 0.002 ≤ *p* ≤ 0.004).

At the after-test, significantly higher speed was found for the control group than both the periodic SA group and the continuous SA group (control group 22.4 mm/s, periodic SA group 8.5 mm/s, continuous SA group 9.8 mm/s, 0.013 ≤ *p* ≤ 0.049). Additionally, the periodic SA group achieved a significantly smoother performance compared to the control group (control group 0.3 mm/s^3^, periodic SA group 0.1 mm/s^3^, *p* = 0.023).

Across all tests, no significant differences were found between groups for handedness (0.285 ≤ *p* ≤ 0.433) and distance between tips of the instruments (0.053 ≤ *p* ≤ 0.797).

All differences in primary and secondary outcome parameters between groups, are shown in Table 1.

**Table 1:** Differences between groups for primary and secondary outcome parameters of laparoscopic suturing

Parameter	Pre-test (Median, IQR)	Baseline-test (Median, IQR)	After-test (Median, IQR)
Time (s)			
Control group	614.0 (827.0)	167.0 (115.0)	178.0 (78.0)
Periodic SA group	692.5 (479)	222.0 (13)	222.0 (64)
Continuous SA group	532.0 (523.0)	220.0 (89.0)	221.0 (102.0)
* p* value	0.362	0.222	0.095
Distance (m)			
Control group	21.1 (35.8)	7.6 (14.2)	12.3 (12.7)
Periodic SA group	20.2 (29.9)	7.1 (15.7)	7.6 (4.4)
Continuous SA group	23.7 (31.4)	8.1 (7.4)	6.8 (17.6)
* p* value	0.934	0.923	0.468
Handedness (%)			
Control group	19.9 (23.9)	17.5 (24.8)	29.9 (24.2)
Periodic SA group	16.1 (16.0)	13.4 (20.7)	20.6 (22.7)
Continuous SA group	23.3 (21.4)	13.2 (25.1)	20.9 (31.2)
* p* value	0.316	0.433	0.285
Off-screen (%)			
Control group	0.9 (5.0)	0.7 (3.4)	2.2 (25.1)
Periodic SA group	0.9 (1.6)	0.5 (2.1)	2.2 (4.7)
Continuous SA group	0.1 (6.9)	0.0 (0.4)	0.4 (3.3)
* p* value	0.372	0.018*	0.076
Speed (mm/s)			
Control group	8.9 (10.7)	13.2 (12.8)	22.4 (33.7)
Periodic SA group	7.3 (10.9)	7.7 (13.7)	8.5 (14.7)
Continuous SA group	9.9 (16.5)	11.1 (9.9)	9.8 (14.4)
* p* value	0.530	0.424	0.033*
Acceleration (mm/s^2^)			
Control group	3.9 (3.2)	5.6 (2.8)	5.3 (1.9)
Periodic SA group	3.7 (2.0)	3.1 (2.8)	3.5 (1.3)
Continuous SA group	3.9 (1.2)	3.6 (3.1)	3.5 (1.3)
* p* value	0.811	0.007*	0.062
Smoothness (mm/s^3^)			
Control group	0.0 (0.1)	0.3 (0.3)	0.3 (0.2)
Periodic SA group	0.0 (0.1)	0.1 (0.1)	0.1 (0.1)
Continuous SA group	0.1 (0.0)	0.1 (0.1)	0.2 (0.1)
* p* value	0.419	0.004*	0.038*
Distance between (cm)			
Control group	5.1 (0.8)	5.9 (1.8)	4.2 (2.5)
Periodic SA group	5.9 (1.8)	5.4 (1.5)	5.3 (1.1)
Continuous SA group	5.6 (2.3)	5.6 (1.7)	5.7 (1.4)
* p* value	0.078	0.797	0.053

*p value < 0.05 is considered significant

### Differences within groups

The control group scored a significant shorter time at the baseline-test and after-test, compared to the pre-test (pre-test 614.0 s, baseline-test 167.0 s, after-test 178.0 s, *p* = 0.001). When comparing distance, a shorter distance was seen at the baseline-test and the after-test as well (pre-test 21.1 m, baseline-test 7.6 m, after-test 12.3 m, 0.001 ≤ *p* ≤ 0.009). In post hoc analysis with a Bonferroni correction applied, no significant difference was seen for speed. When comparing smoothness between all tests, a significant higher smoothness was found at the baseline-test and after-test, compared to the pre-test (pre-test 0.0 mm/s^3^, baseline-test 0.3 mm/s^3^, after-test 0.3 mm/s^3^, *p* = 0.001).

The periodic SA group demonstrated a significantly shorter time at both baseline-test and after-test, compared to the pre-test (pre-test 692.5 s, baseline-test 222.0 s, after-test 222.0 s, *p* = 0.001). Additionally, a shorter distance was observed at the baseline-test and after-test (pre-test 20.2 m, baseline-test 7.1 m, after-test 7.6 m, *p* = 0.006), while significantly higher smoothness was evident at both baseline-test and after-test (pre-test 0.0 mm/s^3^, baseline-test 0.1 mm/s^3^, after-test 0.1 mm/s^3^, *p* = 0.001). Applying a Bonferroni correction in post hoc analysis yielded no significant difference in distance between instrument tips.

The continuous SA group demonstrated a significantly shorter time at the baseline-test as well as the after-test, compared to the pre-test (pre-test 532.0 s, baseline-test 220.0 s, after-test 221.0 s, *p* = 0.001). Regarding distance, a significant difference was found between the pre-test and baseline-test (pre-test 23.7 m, baseline-test 8.1 m, *p* = 0.002). When comparing smoothness across all tests, a significantly higher smoothness was observed in both baseline-test and after-test, compared to the pre-test (pre-test 0.1 mm/s^3^, baseline-test 0.1 mm/s^3^, after-test 0.2 mm/s^3^, 0.001 ≤ *p* ≤ 0.005).

All differences in primary and secondary outcome parameters within groups in different test-moments, are shown in Table 2.

**Table 2:** Differences within groups for primary and secondary outcome parameters of laparoscopic suturing in different test-moments

Parameter	*p* value
Time (s)	
Control group	0.001*
Periodic SA group	0.001*
Continuous SA group	0.002*
Distance (m)	
Control group	0.002*
Periodic SA group	0.002*
Continuous SA group	0.012*
Handedness (%)	
Control group	0.150
Periodic SA group	0.465
Continuous SA group	0.465
Off-screen (%)	
Control group	0.611
Periodic SA group	0.229
Continuous SA group	0.465
Speed (mm/s)	
Control group	0.040*
Periodic SA group	0.589
Continuous SA group	0.327
Acceleration (mm/s^2^)	
Control group	0.128
Periodic SA group	0.790
Continuous SA group	0.943
Smoothness (mm/s^3^)	
Control group	0.001*
Periodic SA group	0.001*
Continuous SA group	0.002*
Distance between (cm)	
Control group	0.504
Periodic SA group	0.028*
Continuous SA group	0.838

*p value < 0.05 is considered significant

## Discussion

No significant differences were observed in outcome parameters of laparoscopic suturing between participants who engaged in self-assessment and reflection before practice solely at test-moments and those who used self-assessment and reflection before practice in a higher frequency, namely prior to every training- and test-moment. Thus, a higher frequency of self-assessment in laparoscopic suturing did not appear to offer additional benefits compared to a more limited frequency of self-assessment.

At the pre-test, no significant differences were observed among the three groups. Despite incorporating reflection before practice in the periodic SA group and the continuous SA group, these groups did not outperform the control group at the start of their learning curve, unlike prior research on self-assessment in open surgery [17]. Additionally, primary outcome parameters time and distance showed no significant differences between groups at the baseline-test and after-test.

However, the continuous SA group had a significantly lower off-screen percentage compared to the control group at the baseline-test. The parameter off-screen is clinically relevant, as instruments out of sight increase the risk of causing tissue damage. Additionally, at the after-test, the control group scored a higher speed of instruments compared to both self-assessment groups. Despite this higher speed, the control group did not achieve a faster time in executing the suturing task, indicating less efficiency in instrument movements. This inefficiency was further supported by worse results for acceleration and smoothness at the baseline-test in the control group, compared to both self-assessment groups. At the after-test, the control group also scored significantly worse in smoothness than the periodic SA group. These differences may be attributed to more deliberate execution following reflection before practice, resulting in slower and smoother movements.

The mentioned differences in secondary outcome parameters confirm previous research demonstrating that self-assessment enhances overall performance in laparoscopic surgery by fostering a better understanding of strengths and weaknesses [14, 25]. This study aimed to investigate the optimal frequency of self-assessment during a laparoscopic training schedule. A lower frequency (three times) of self-assessment provided similar benefits as a higher frequency (thirteen times) during the laparoscopic training schedule. Self-assessment and reflection before practice, when done just once, seemed to have no immediate effect, as no differences were seen at the pre-test. However, the potential impact later in the learning curve of a self-assessment frequency of one, has not been investigated. Further research should explore whether the use of self-assessment solely on the start of the learning curve influences later stages of the learning process.

Laparoscopic suturing is a crucial component of laparoscopic surgical training, however, acquiring and retaining this specific skill is very difficult [5–8]. Therefore, innovative approaches must be explored to develop and retain these skills. Laparoscopic suturing skills improve through the implementation of verbal feedback, video feedback, and self-assessment [16]. Previous research has demonstrated that self-assessment can add significant value during laparoscopic simulation training [14, 16, 25]. However, the optimal frequency for self-assessment, remained unknown.

This study demonstrates that incorporating self-assessment and reflection before practice improves safety and efficiency of laparoscopic suturing. However, it also suggests that the frequency of self-assessment, whether high or low, does not necessary translate into additional advancements. From a learning efficiency perspective, a lower frequency of self-assessment appears to be optimal.

### Limitations

A few limitations of this study need to be addressed. At the start of the study, participants reported their surgical experience and no significant differences were found between groups. However, participant’s fine motor skills were not assessed. Consequently, a potential difference in the fine motor skills level between groups, and therefore bias at the starting level of participants, potentially exists.

In this study, objective parameters were used as primary and secondary outcome parameters. While these objective parameters can provide an indication of skills level, as investigated before, they may not necessarily reflect the quality of the laparoscopic sutures [26, 27]. Ideally, the quality of the laparoscopic sutures should also be assessed. Further research could explore the correlation between objective parameters and expert assessment.

## Conclusion

A higher frequency of self-assessment did not result in a shorter time or more efficient suturing. However, the use of self-assessment and reflection before practice in laparoscopic suturing training proves to be beneficial to the percentage out of screen, as well as adequate acceleration and smoothness during the execution of a laparoscopic suturing task, compared to not using self-assessment and reflection before practice. Interestingly, a lower frequency of self-assessment and reflection before practice seems to be optimal for maximizing self-assessment efficiency.
